# Evaluation of Multiple-Locus Variable Number of Tandem Repeats Analysis for Typing Livestock-Associated Methicillin-Resistant *Staphylococcus aureus*

**DOI:** 10.1371/journal.pone.0054425

**Published:** 2013-01-21

**Authors:** Karin M. Brandt, Alexander Mellmann, Britta Ballhausen, Christian Jenke, Peter J. van der Wolf, Els M. Broens, Karsten Becker, Robin Köck

**Affiliations:** 1 Institute of Hygiene, University Hospital Münster, Münster, Germany; 2 Institute of Medical Microbiology, University Hospital Münster, Münster, Germany; 3 Pig Health Department, Animal Health Service (AHS), Deventer, The Netherlands; 4 Veterinary Microbiological Diagnostic Centre, Department of Infectious Diseases and Immunology, Faculty of Veterinary Medicine, Utrecht University, Utrecht, The Netherlands; Rockefeller University, United States of America

## Abstract

**Background:**

The increasing occurrence of livestock-associated (LA) methicillin-resistant *Staphylococcus aureus* (MRSA) associated with the clonal complex (CC) 398 within the past years shows the importance of standardized and comparable typing methods for the purposes of molecular surveillance and outbreak detection. Multiple-locus variable number of tandem repeats analysis (MLVA) has recently been described as an alternative and highly discriminative tool for *S. aureus*. However, until now the applicability of MLVA for the typing of LA-MRSA isolates from different geographic origin has not been investigated in detail. We therefore compared MLVA and *S. aureus* protein A (*spa*) typing for characterizing porcine MRSA from distinct Dutch and German farms.

**Methodology/Principal Findings:**

Overall, 134 MRSA isolates originating from 21 different pig-farms in the Netherlands and 36 farms in Germany comprising 21 different *spa* types were subjected to MLVA-typing. Amplification and subsequent automated fragment sizing of the tandem repeat loci on a capillary sequencer differentiated these 134 isolates into 20 distinct MLVA types. Whereas overall MLVA and *spa* typing showed the same discriminatory power to type LA-MRSA (p  = 0.102), MLVA was more discriminatory than *spa* typing for isolates associated with the prevalent *spa* types t011 and t034 (Simpson’s Index of Diversity 0.564 vs. 0.429, respectively; p<0.001).

**Conclusion:**

Although the applied MLVA scheme was not more discriminatory than *spa* typing in general, it added valuable information to *spa* typing results for specific *spa* types (t011, t034) which are highly prevalent in the study area, i.e. Dutch-German border area. Thus, both methods may complement each other to increase the discriminatory power to resolute highly conserved clones such as CC398 (*spa* types t011, t034) for the detection of outbreaks and molecular surveillance of zoonotic MRSA.

## Introduction

Methicillin-resistant *Staphylococcus aureus* (MRSA) have emerged in livestock within the past years and are increasingly detected in humans [1], [2], [3], [4]. Predominantly, livestock-associated MRSA (LA-MRSA) isolates belong to a distinct MRSA lineage characterized by the clonal complex (CC) 398 as defined by multilocus sequence typing (MLST) [3].

Livestock animals are usually only colonized with LA-MRSA CC398. However, this clonal lineage has occasionally been identified as the causative agents for wound infections in horses [5] or mastitis in cows [6]. In pigs, LA-MRSA has been associated with pathological lesions, abortion and systemic diseases [7], [8].

The impact of LA-MRSA CC398 on humans has been determined in many epidemiological studies. Direct contact to livestock is a significant risk factor for colonization with such strains leading to nasal MRSA carriage in 29% [4] to 86% [9] of pig farmers and 4.6% [10] to 45% [9] of veterinarians. Especially in areas with a high density of livestock farming, MRSA CC398 represents a significant proportion among the MRSA isolates derived from hospital inpatients [11]. Moreover, healthcare-associated outbreaks of MRSA CC398 have already been documented [12], [13]. MRSA CC398 has been discussed to be less virulent than hospital- or community-acquired MRSA [14]. Nevertheless, MRSA CC398 has shown its potential to cause human infections (e.g. pneumonia, otomastoiditis, endocarditis and bacteraemia) [15], [16].

To elucidate the molecular epidemiology of LA-MRSA CC398 and to discriminate sporadic from outbreak related cases, different typing techniques have been proposed [17], [18]. However, the lack of a standardized and feasible method with a high epidemiological resolution still hampers studies on outbreaks and transmission routes. MRSA CC398 cannot be typed by standard protocols for pulsed-field gel electrophoresis (PFGE), which separates fragments after *Sma*I macrorestriction [19]. Although PFGE approaches using *Cfr*9I as alternative restriction enzyme have been successfully applied [18], [20], these methods are laborious and expensive. Furthermore, PFGE can be problematic due to intra- and inter-laboratory reproducibility aspects. Sequence-based typing of the *S. aureus* protein A gene (*spa*) is feasible, cheaper and ensures comparability [21], [22]. However, LA-MRSA CC398 isolates are mostly associated with a very limited number of *spa* types including t011 and t108 as prevalent clones in the Netherlands [23] and t011, t034 and t2510 as prevalent clones in Germany [24].

A recent study by Schouls et al. has compared PFGE, MLST and *spa* typing with a new high throughput typing approach using multiple-locus variable number of tandem repeat analysis (MLVA) [17]. This technique yielded a high discriminatory power and a high congruence with PFGE, MLST and was twice as discriminatory as *spa* typing for hospital- and community-acquired MRSA. However, particularly for LA-MRSA CC398 isolates, MLVA grouped them into two dominant complexes only and did not increase the discriminatory power in comparison with *spa* typing. Yet, the lack of epidemiological data within the study from Schouls et al. limited its conclusion whether MLVA is suitable for differentiation of LA-MRSA of CC398. Moreover, the study did not include isolates belonging to the *spa* type t034 [17], which is the second most frequent *spa* type associated with LA-MRSA CC398 in northwestern Germany [24], [25].

Therefore, we investigated a diverse collection of LA-MRSA, mostly belonging to the CC398 lineage, from geographical distinct farms and regions in Germany and the Netherlands using the MLVA scheme by Schouls et al. The results of our study shall answer the question whether MLVA can differentiate between LA-MRSA isolates associated with the same *spa* types but obtained in different regions and thereby specifies the information obtained by *spa* typing.

## Materials and Methods

### Selection of Bacterial Strains

All samples were derived from pigs (nasal swabs) of 21 Dutch and 36 German farms located in different geographic regions. They were collected within the framework of the INTERREG IVa project SafeGuard (Workpackage 2.3, MRSA vet-net, III-2-03 = 025). All isolates were initially characterized by *S. aureus* protein A (*spa*) sequence-based typing as described elsewhere [22], [26]. Cluster analysis of *spa* types using the Based Upon Repeat Pattern (BURP) algorithm of the Ridom StaphType software (Ridom GmbH, Münster, Germany) with default parameters as recommended previously was used to assign *spa* types into *spa*-clonal complexes (spa-CC) [26], [27], [28]. Due to the high concordance of *spa* typing and MLST [29], *spa* types clustering with t011, t034 and t108, which have previously been shown to belong to the MLST clonal lineage CC398 were suspected to be part of CC398. For the remaining *spa* types, MLST types were assigned by comparison with data from the *Spa*-server (http://spaserver.ridom.de/) and by literature search.

If available, two MRSA isolates of each *spa* type were chosen from each farm and subjected to MLVA typing. Altogether, we analysed 134 MRSA isolated between 2009 and 2011. Among these, 66 isolates were from Dutch and 68 from German farms.

### Bacterial Growth and DNA Extraction

Confirmed MRSA cultures stored at −80°C were grown overnight on Columbia blood agar plates (Heipha; Eppelheim, Germany) at 37°C in ambient atmosphere [24]. One colony was dispended in 100µl Chelex-100 solution (Bio-Rad, Hercules, CA, USA). After the heating process (10 min at 95°C), the samples were vortexed thoroughly and centrifuged at 13400 g for 3 min. The DNA-containing supernatant was either used directly or stored at −20°C until use in PCR.

### MLVA

MLVA was performed as previously described by Schouls et al. [17] with some modifications. Two multiplex PCRs were prepared. Each multiplex mixture contained four different fluorescently labelled primer sets (Applied Biosystems, Foster City, CA, USA). The primer concentrations in Mastermix 1 were 0.4µM VNTR09_01f-6-FAM, VNTR09_01r, VNTR61_01f-NED, VNTR61_01r, VNTR61_02r, VNTR67_01_f-PET and VNTR67_01r and 0.2 µM VNTR61_02f-VIC and VNTR61_02f. In Mastermix 2, primer concentrations were 0.4 µM VNTR21_01f-VIC, VNTR21_01r, VNTR81_01f-NED and VNTR81_01r and 0.6 µM VNTR24_01f-PET, VNTR24_01r, VNTR63_01f-6-FAM and VNTR63_01. Both PCRs contained a Type-it Multiplex PCR Master Mix (Qiagen, Hilden, Germany) and 2 µl DNA template in a final volume of 25 µl. Amplification was performed using the following program: initial denaturation (5 min at 95°C), 28 cycles of amplification (30 sec at 95°C, 90 sec at 60°C and 30 sec at 72°C) followed by a final step of 30 min at 60°C.

After PCR, samples were diluted 1∶100 in water. 1.5 µl of the diluted samples were mixed with 15 µl of 1200 LIZ size standard (Applied Biosystems) diluted 1∶100 in HPLC-water. To denature the DNA, samples were incubated for 5 min at 95°C and then immediately frozen at −20°C for ≥3 min. Fragments were separated on an ABI Prism 3130xl Genetic Analyzer System (Applied Biosystems). The results were analysed in the GeneMapper 4.0 software (Applied Biosystems) to calculate the correct number of repeats for each VNTR locus. To calibrate sequencer-specific variation in fragment lengths, the number of repeats of reference strain *S. aureus* N315 strain was initially determined *in silico* on the basis of its genome sequence (reference sequence NC_002745; National Center for Biotechnology Information, Bethesda, MD, USA) in accordance with the MLVA protocol provided by the National Institute of Public Health and the Environment of the Netherlands (RIVM) on the MLVA home page (http://www.mlva.net/saureus/default.asp). The lengths of the determined repeats were subtracted from the fragment length of each particular locus detected in independent runs on the Genetic Analyzer. The resulting offset-size (primer plus VNTR-flanking regions) was used to create the respective bin used in the GeneMapper software. In each run, the reference strain was included to assure correct repeat determination of unknown isolates. Partial repeats were rounded to the nearest repeat number.

If a VNTR locus was not detected during fragment analysis, PCR for that particular locus was repeated in a singleplex reaction with the same primer concentrations as in the multiplex-PCR. In rare cases, so-called stutter-peaks (peaks that are typically ≤1 repeat length shorter than the main peak) occurred. In that particular instance, the peak with the highest fluorescent level was used to calculate the repeat number.

### Data Analysis

The geographical map and the minimum spanning tree were created with the Ridom MLVA Compare software version 0.28 (Ridom GmbH, Münster, Germany). MLVA profiles were entered into the MLVA home page (http://www.mlva.net/saureus/default.asp) of the RIVM to query the corresponding MLVA-types and complexes. To calculate the discriminatory power and concordance of the typing methods we used EpiCompare software version 1.0 (Ridom GmbH, Münster, Germany) and an internet tool (http://darwin.phyloviz.net/ComparingPartitions/index.php?link=Home).

## Results

Overall, MLVA was successfully performed on 133 isolates comprising 20 different *spa* types (Table 1). The most frequent *spa* types were t011 (42.5%, n = 57), t034 (18.7%, n = 25) and t108 (14.9%, n = 20). For one isolate from the Netherlands (*spa* type t151), none of the eight VNTR-loci was amplified after repeated PCR. Therefore, no MLVA-profile could be determined and this isolate was excluded from further analyses.

**Table 1:** Distribution of *spa* and MLVA types among the analysed MRSA isolates.

*Spa* type	*Spa*-CC	Presumptive MLST CC	MLVA type	MLVA-complex	No. of isolates	No. of farms^i^
t011	011	398^a^	398	398	52	33
t011	011	398^a^	3750	NA^h^	1	1
t011	011	398^a^	2215	398	1	1
t011	011	398^a^	555	398	2	2
t011	011	398^a^	713	398	1	1
t108	011	398^a^	572	398	20	12
t034	011	398^a^	569	398	14	10
t034	011	398^a^	588	398	8	5
t034	011	398^a^	591	398	2	1
t034	011	398^a^	556	398	1	1
t6320	011	398^b^	569	398	3	2
t2346	011	398^b^	565	398	4	2
t1451	011	398^a^	572	398	2	2
t7621	011	398^b^	1283	398	2	1
t1250	011	398^b^	590	398	1	1
t1184	011	398^b^	567	398	1	1
t1606	011	398^b^	572	398	1	1
t3423	011	398^b^	572	398	1	1
t899	singleton	398^a^; 9^c^	567	398	3	2
t899	singleton	398^a^; 9^c^	398	398	1	1
t5838	singleton	NA^h^	565	398	2	1
t2510	excluded	398^a^	1845	398	3	2
t1456	excluded	398^d^	566	398	1	1
t2383	excluded	398^e^	568	398	1	1
t1344	excluded	398^d^	1845	398	1	1
t002	singleton	5^c^	31	5	2	1
t015	singleton	45^f^	45	45	1	1
t127	singleton	5^c^; 1^g^	1727	1	1	1
t151	singleton	NA^h^	NT	NT	1	1

a according to Köck et al. (2009) [24].

b as determined by the Based Upon Repeat Pattern (BURP) algorithm.

c according to Hasman et al. (2010) [30].

d according to European Food Safety Authority (2009) [25].

e according to Graveland et al. [32].

f according to the Ridom SpaServer.

g according to Mellmann et al. (2008) [27].

h not available.

i number of farms from which isolates of the respective *spa* type have been derived and included.

**Figure 1:**
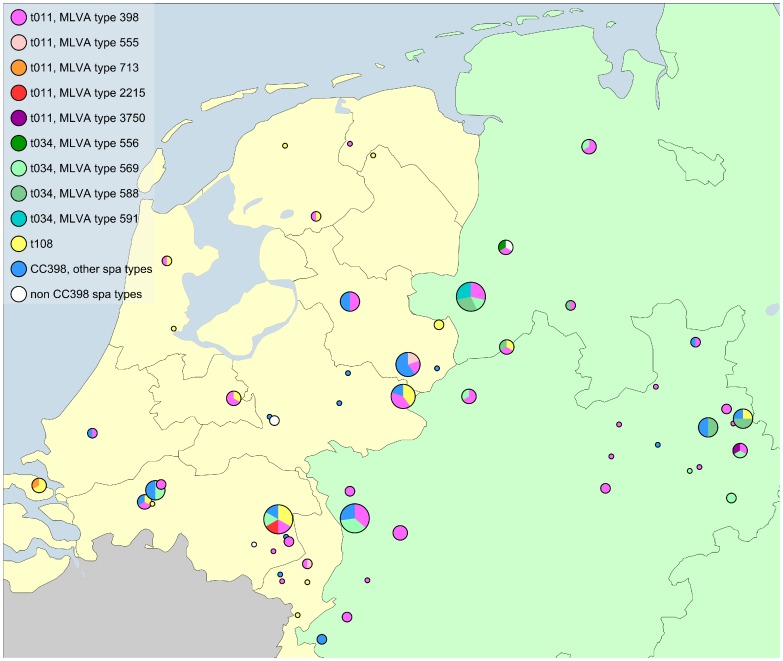
Geographic origin of the analysed isolates in Germany and the Netherlands. One dot represents farms in one same postal code area; the size of a dot is proportional to the amount of isolates obtained from these farms. The proportion of different *spa* types (and MLVA types for the predominant t011 and t034 *spa* types) is indicated by different colours.

Among the 133 MLVA-typeable associated with 20 different *spa* types, MLVA resolved 20 different MLVA types. MLVA was able to discriminate isolates that were indistinguishable by *spa* typing, by distinguishing five different MLVA subtypes among *spa* type t011, four among *spa* type t034 and two among *spa* type t899, respectively. The *spa* type t108 was represented by only one MLVA type (type 572). Each of the remaining *spa* types t2510, t2346, t5838, t015, t1250, t1456, t2383, t6320, t002, t1451, t7621, t1184, t127, t1344, t1606 and t3423 was also characterized by solely one MLVA-type (Table 1). An overview of the geographic location of the isolates’ origin as well as the distribution of the *spa* types and main MLVA types is given in Fig. 1.

**Figure 2:**
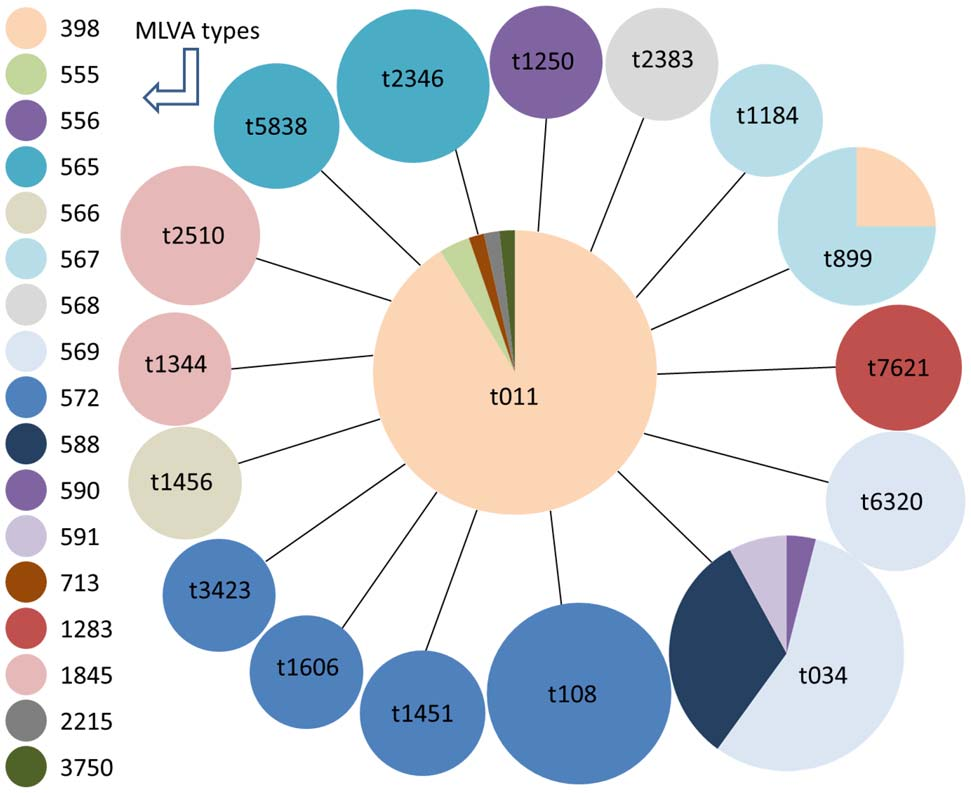
Minimum spanning tree based on *spa* types for all isolates associated with LA-MRSA CC398. Each dot represents a different *spa* type with the diameter of a dot being proportional to the quantity of the isolates included. Different colours within the dots indicate different MLVA types. The *spa* types that are associated with the same MLVA type are identically coloured.

Of the 20 different *spa* types, 17 were assigned to CC398 according to clustering by the BURP algorithm, MLST and MLVA data (Table 1). An overview of the MLVA-types that were determined for isolates of the CC398 lineage is shown in Fig. 2. Importantly, fig. 2 shows that *spa* type t034 showed the greatest diversity with MLVA types 556, 569, 588 and 591 accounting for 4%, 56%, 32% and 8% of all t034 isolates, respectively. The distribution of MLVA types within *spa* type t011 was as follows: MLVA type 398 represented 91% of all isolates, type 555 4% and MLVA types 713, 2215 and 3750 each 2%, respectively.

The calculation of the Simpson’s index of diversity (*ID*) for all isolates characterized yielded similar *ID*s of 0.789 and 0.760 for by *spa* typing and MLVA, respectively (Table 2). Therefore, both methods had the same discriminatory power to distinguish between LA-MRSA isolates (p  = 0.102). The adjusted Rand’s coefficient (AR; 95% confidence interval) was 0.790 (0.696–0.889).

**Table 2:** Simpson’s Indices of Diversity (*ID*) and concordance of MLVA and *spa* typing for all analysed isolates, for isolates associated with CC398 and for isolates associated with the two predominant *spa* types associated with CC398.

Typing method	No. of types	Simpson’s ID (C.I 95%)^1^	Adjusted Wallace’s coefficient^1^	
**All analysed isolates**			***spa***	**MLVA**
MLVA	20	0.789 (0.735–0.844)	0.863(0.775–0.950)	NA
*spa*	20	0.760 (0.701–0.818)	NA	0.728(0.598–0.859)
**All ** ***spa*** **-types associated with CC398**			***spa***	**MLVA**
MLVA	17	0.776 (0.720–0.833)	0.860(0.770–0.949)	NA
*spa*	17	0.745 (0.685–0.805)	NA	0.724(0.591–0.856)
***Spa*** **-types t011 and t034**			***spa***	**MLVA**
MLVA	9	0.564 (0.452–0.677)	1.000(1.000–1.000)	NA
*spa*	2	0.429 (0.350–0.508)	NA	0.580(0.378–0.783)

1 C. I. = confidence interval; NA = not applicable.

Since we were aiming to reveal the discriminatory power of MLVA to distinguish in particular among MRSA isolates associated with CC398, we repeated the calculation but included only the *spa* types (t011, t034, t108, t899, t1184, t1250, t1344, t1451, t1456, t1606, t2346, t2383, t2510, t3423, t5838, t6320 and t7621) that are indicative for this complex according to BURP clustering, MLST; MLVA data and literature [24], [25], [27], [30], [31], [32]. The exclusion of all non-CC398 *spa* types did not reveal a significant difference in the discriminatory power of both methods (MLVA: *ID*  = 0.776; *spa* typing: *ID*  = 0.745; p  = 0.102). The AR was 0.786 (0.691–0.886).

To evaluate the applicability and additional value of MLVA compared to *spa* typing for outbreaks caused by the most prevalent *spa*-types associated with MRSA CC398, we therefore concentrated on the predominant *spa* types t011 and t034 among porcine isolates detected within the INTERREG IVa project SafeGuard and excluded all others from the calculation. This resulted in an increase of the discriminatory power of MLVA (*ID*  = 0.564) compared to *spa* typing (*ID*  = 0.429; p<0.001). As documented by the adjusted Wallace’s coefficients, in this calculation MLVA added valuable information to *spa* typing, since isolates being associated with one same *spa* type, had a probability of 0.58 (58%) to have the same MLVA type only. The AR was 0.734 (0.605–0.866).

## Discussion

In this study, we evaluated a previously described MLVA scheme [17] to type LA-MRSA. Overall, we found that nearly all (133 of 134) MRSA isolates were typeable by MLVA (99.3%). The failed typing of one isolate might be due to modified DNA or chromosomal variability in *S. aureus* that leads to the absence or the mutation of the primer targets [33].

Schouls et al. [17] have recently shown that typing by MLVA reveals the same degree of differentiation as *spa* sequencing regarding LA-MRSA isolates. This finding was in contrast to other publications which had shown that MLVA schemes are usually more discriminatory compared to *spa* typing [33], [34]. In order to rule out the possibility that the lacking ability of MLVA to discriminate between LA-MRSA strains observed in the study by Schouls et al. [17] was due to a selection bias caused by the inclusion of epidemiologically related MRSA isolates, we have systematically chosen isolates from different locations (57 different farms in Germany and the Netherlands) and subjected them to MLVA typing. We hypothesized that typing of geographically unrelated isolates would increase the discriminatory power. As a result, we showed that even when the geographical diversity and epidemiological differentiation were ensured, MLVA was not more discriminatory than *spa* typing. As MRSA has only evolved a few decades ago, this might reflect the monomorphic background of this pathogen [35].

However, we found that for specific *spa* types, MLVA typing enabled to separate isolates that were indistinguishable by *spa* typing. Isolates that are associated with the highly prevalent *spa* types t011 and t034 were each diversified into more than one MLVA type (t011 in 5 and t034 in 4 MLVA types, respectively). While for t011, performing MLVA in addition to *spa* was of limited value, because 91% of all t011 isolates were associated with MLVA type 398, isolates belonging to *spa* type t034 demonstrated a higher diversity. This finding is of importance, because t034, which is a major *spa* type among European livestock [25], was not included in previous analysis [12]. This result has practical applications both for tracing LA-MRSA among livestock and for human medicine where in some regions, farmers (colonized with LA-MRSA CC398 in up to 77%) introduce these MRSA strains into regional hospitals which could facilitate nosocomial spread [11], [32], [36]. We therefore suggest that additional MLVA typing of LA-MRSA isolates assigned to *spa* types t034 and (sometimes) t011 can add valuable information to characterize LA-MRSA for example in healthcare-associated outbreaks. In contrast, for isolates associated with *spa* type t108, which is highly prevalent in the Netherlands [37], or the other *spa* types included in this study, additional MLVA typing did not reveal a higher degree of differentiation.

Currently, *spa* typing is a cheap and feasible method that is widely used for characterizing MRSA. Furuya et al. suggested in a recent study that for best resolution of the results, *spa* typing should be used in combination with MLVA or PFGE for short-term evolutionary studies [38]. Our results support this opinion, at least for certain *spa* types. Since hitherto no standardized and widely used protocol for MLVA typing exists, we suggest that *spa* typing of LA-MRSA should be performed to basically characterize MRSA CC398 isolates. Additional MLVA typing of isolates assigned to *spa* types t011 or t034 might then lead to a more detailed characterization, if this is needed for case tracing, outbreak detection, or investigations into the pathways of transmission of MRSA CC398 in healthcare facilities. In the veterinary field, MLVA typing could increase the resolution and thereby contribute to a more precise understanding of the distribution of LA-MRSA by national and international pig trading or environmental spread [39], [40], [41].

An advantage of MLVA compared to *spa* typing is that it is faster and cheaper to perform. Especially in those laboratories with capillary sequencing capacities both methods could complement each other, although the recent description of multiple different MLVA schemes [17], [42], [43], [44], [45] stresses the need to agree upon the use of concerted protocols when this technique is increasingly applied.

While in this study MLVA typing was not shown to be more discriminatory than single locus *spa* typing in general, this approach was able to subdivide LA-MRSA isolates of the highly prevalent *spa* types t011 and t034. Thus, in areas with a high prevalence of LA-MRSA CC398, MLVA typing offers a useful additional tool for epidemiological studies and MRSA surveillance purposes.
